# The potential of data linkage for improving social care provision

**DOI:** 10.23889/ijpds.v8i2.2298

**Published:** 2023-09-14

**Authors:** Magdalena Rossetti, Rick Hood

**Affiliations:** 1 Policy in Practice, London, United Kingdom; 2 LSE, London, United Kingdom; 3 Kingston University, Kingston upon Thames, United Kingdom

## Objectives

The main objective of the project was to link case-level data from Children’s Social Care (CSC) service with household-level data on means-tested benefits, in order to analyse the dynamic relationship between families’ financial situation and the demand for CSC services at the household level.

## Methods

The study was a secondary quantitative analysis of administrative data from one local authority in England. After completing research ethics review and data governance procedures, monthly data on families receiving housing benefits and council tax benefits payments were linked to child-level data on referrals to CSC services over a two-year period. The match was carried out based on personal identifiers, and once the linkage process was complete, a pseudonymised linked dataset (containing no personal identifiers) was used for all subsequent analyses.

## Results

We find that it is feasible to link children’s social care and benefits data. Our findings demonstrate a significant overlap between households receiving means-tested benefits and those referred to CSC services, underscoring the fact that most referrals involve low-income families. Our study further indicates that the children referred to CSC services primarily reside in deprived areas characterized by limited access to housing and services, as well as poor housing conditions. Additionally, we observed that children in households experiencing financial strain are twice as likely to be referred to CSC services.

## Conclusion

Linking benefits data with CSC referrals can shed light on important questions related to the relationship between poverty and demand for child welfare services. For example, mechanisms through which poverty drives demand for child welfare services, including the role of persistent poverty, financial precarity, reductions or disruptions to benefits payments, unemployment, overcrowding, rent increases, evictions, etc.

